# NADPH oxidase in brain injury and neurodegenerative disorders

**DOI:** 10.1186/s13024-017-0150-7

**Published:** 2017-01-17

**Authors:** Merry W. Ma, Jing Wang, Quanguang Zhang, Ruimin Wang, Krishnan M. Dhandapani, Ratna K. Vadlamudi, Darrell W. Brann

**Affiliations:** 1 Charlie Norwood VA Medical Center, One Freedom Way, Augusta, GA 30904 USA; 2 Department of Neuroscience and Regenerative Medicine, Medical College of Georgia, 1120 Fifteenth Street, Augusta, GA 30912 USA; 3 Department of Neurosurgery, Medical College of Georgia, Augusta University, 1120 Fifteenth Street, Augusta, GA 30912 USA; 4 Department of Obstetrics and Gynecology, University of Texas Health Science Center, 7703 Medical Drive, San Antonio, TX 78229 USA

**Keywords:** NADPH oxidase, Alzheimer’s disease, Stroke, Neurodegeneration, Oxidative stress, Traumatic brain injury, Parkinson’s disease, Amyotrophic lateral sclerosis, Huntington’s disease, Multiple sclerosis

## Abstract

Oxidative stress is a common denominator in the pathology of neurodegenerative disorders such as Alzheimer’s disease, Parkinson’s disease, Huntington’s disease, amyotrophic lateral sclerosis, and multiple sclerosis, as well as in ischemic and traumatic brain injury. The brain is highly vulnerable to oxidative damage due to its high metabolic demand. However, therapies attempting to scavenge free radicals have shown little success. By shifting the focus to inhibit the generation of damaging free radicals, recent studies have identified NADPH oxidase as a major contributor to disease pathology. NADPH oxidase has the primary function to generate free radicals. In particular, there is growing evidence that the isoforms NOX1, NOX2, and NOX4 can be upregulated by a variety of neurodegenerative factors. The majority of recent studies have shown that genetic and pharmacological inhibition of NADPH oxidase enzymes are neuroprotective and able to reduce detrimental aspects of pathology following ischemic and traumatic brain injury, as well as in chronic neurodegenerative disorders. This review aims to summarize evidence supporting the role of NADPH oxidase in the pathology of these neurological disorders, explores pharmacological strategies of targeting this major oxidative stress pathway, and outlines obstacles that need to be overcome for successful translation of these therapies to the clinic.

## Background

Neurodegenerative disorders such as stroke, traumatic brain injury (TBI), Alzheimer’s disease (AD), Parkinson’s disease (PD), Huntington’s disease (HD), amyotrophic lateral sclerosis (ALS), and multiple sclerosis (MS) carry substantial disease burden, not only in terms of health suffering but also in economic costs [1–6]. Although the clinical manifestations differ for these disorders, a common denominator in their pathology is the induction of oxidative stress [7, 8]. For the past several decades, evidence has accumulated supporting oxidative stress as an underlying common denominator of brain injury and neurodegenerative disorders. For instance, a keyword search on PubMed using the terms “oxidative stress and neurodegeneration” or “oxidative stress and brain injury” yielded over 8,400 citations, with approximately half of these occurring in the last five years. From this body of research, it has become clear that pathological conditions such as ischemia, trauma, and neurodegenerative processes, markedly enhances generation of ROS in the brain [9, 10]. Therapies attempting to target oxidative stress have typically focused on compounds that scavenge the free radicals to eliminate them from the system. Unfortunately, these therapies have generally been unsuccessful in clinical trials despite promise in animal models.

The lack of success to date of clinical trials using ROS scavenging drugs has led to the hypothesis that inhibition of the *generation*, rather than the *scavenging*, of ROS may be a more successful avenue of therapy. Correlative to this hypothesis, many laboratories have focused on enhancing our understanding of the role of ROS-generating pathways in various pathological situations. These studies have led to the identification of the enzyme, nicotinamide adenine dinucleotide phosphate (NADPH) oxidase as a major generator of ROS that contributes to the pathology of both acute brain injury and chronic neurodegenerative disorders [11, 12]. While NADPH oxidase undoubtedly contributes to *physiological* processes in the brain [13, 14], the primary goal of the current review is to summarize the evidence supporting a role of NADPH oxidase following acute ischemic and traumatic brain injury, as well as in chronic neurodegenerative disorders, and explore the potential efficacy and strategies for therapeutic targeting of this major ROS-generating pathway.

### Origin and cellular consequence of oxidative stress in brain injury and neurodegenerative disorders

**Fig. 1:**
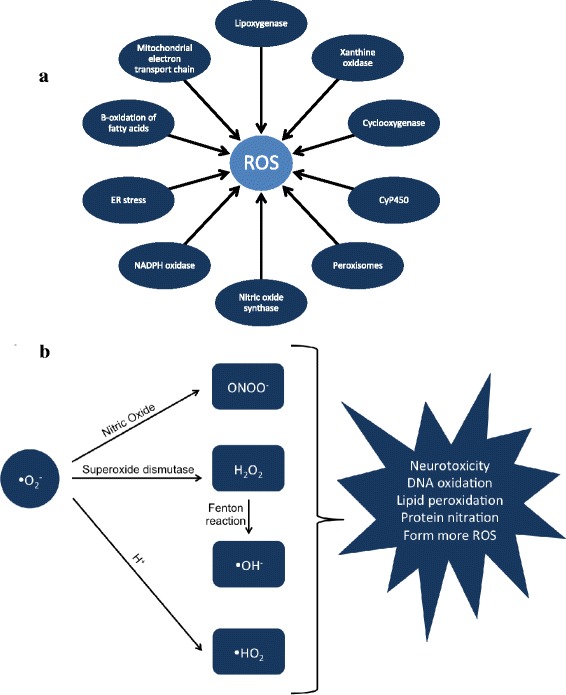
**a** Cellular Sources of ROS. ROS can be generated intracellularly via the action of various organelles, enzymes, and processes. **b** Conversion of Superoxide to Secondary ROS. Once produced, superoxide can interact with various molecules to generate secondary radicals. Superoxide can react with nitric oxide to produce peroxynitrite. Superoxide dismutase can also convert superoxide into hydrogen peroxide, which can then undergo a Fenton reaction to produce hydroxyl radicals and ions. Interaction of superoxide with protons can produce hydroperoxyl radicals. These secondary radicals are highly reactive and can be neurotoxic via alterations of macromolecules and amplification of oxidative stress

ROS are generated endogenously from molecular oxygen by cellular oxidases, mono- and di-oxygenases of the mitochondrial electron chain transport system, or peroxidases. The major ROS involved in oxidative stress include superoxide anion, hydrogen peroxide, and hydroxyl radicals [15]. There are also reactive nitrogen species (RNS) such as nitric oxide and peroxynitrite that participate in oxidative stress in the CNS and throughout the body [10, 16]. As shown in Fig. 1a, there are multiple sources and processes that lead to generation of superoxide and ROS in cells. Superoxide, once produced, can cause oxidative damage directly, or it can interact with other molecules to generate “secondary” radicals via enzymatic or metal-catalyzed reactions. Along these lines, as shown in Fig. 1b, superoxide can react with nitric oxide to generate the RNS peroxynitrite, which is highly reactive, and when over-produced can deleteriously nitrate most biological molecules in all parts of the body, including the brain [16, 17]. Superoxide can also undergo dismutation via superoxide dismutase to form oxygen and hydrogen peroxide [18]. Hydrogen peroxide is highly permeable through cell membranes and can directly damage cells through oxidation of deoxyribonucleic acid (DNA), proteins, and lipids. In the presence of transition metals, hydrogen peroxide can generate highly reactive hydroxyl radicals, which can damage DNA and other molecules in the cell [19, 20]. In addition to modifying and damaging macromolecules, the unpaired electron in ROS can also amplify oxidative damage by generating more free radicals [21]. Finally, ROS-induced modifications can target cysteine residues or oxidize thiols present in protein components of cell signaling [22], and this alteration in signaling can lead to a variety of downstream effects involving gene expression and apoptosis.

The relationship between the cellular consequences of oxidative stress and the progression of brain injury and neurodegenerative disorders has undergone continued refinement. Extensive research has shown that mitochondria-derived ROS are clinically relevant as an important contributing factor for neurodegeneration in brain injury and chronic neurodegenerative disorders. While coverage of this topic is beyond the scope of this review, the reader is referred to several excellent reviews written previously on this subject [23–26]. Recently, there has been increased interest in other sources of ROS, such as NADPH oxidase, nitric oxide synthase, cytochrome450 (cyp450), cyclooxygenase, lipooxygenase, and xanthine oxidase [19, 27]. Of the above list, only NADPH oxidase has as its primary function the generation of ROS, as the others generate ROS as a byproduct [28–30]. Previous reviews have summarized evidence of redox crosstalk between mitochondria and NADPH oxidase that suggests mitochondria may be a key player and trigger in amplifying the burden of oxidative stress [31–33]. In the sections below, we will focus upon evidence concerning the biology, distribution and role of the various NADPH oxidase family members in brain injury and neurodegenerative disorders.

### NADPH oxidase – an overview

**Fig. 2:**
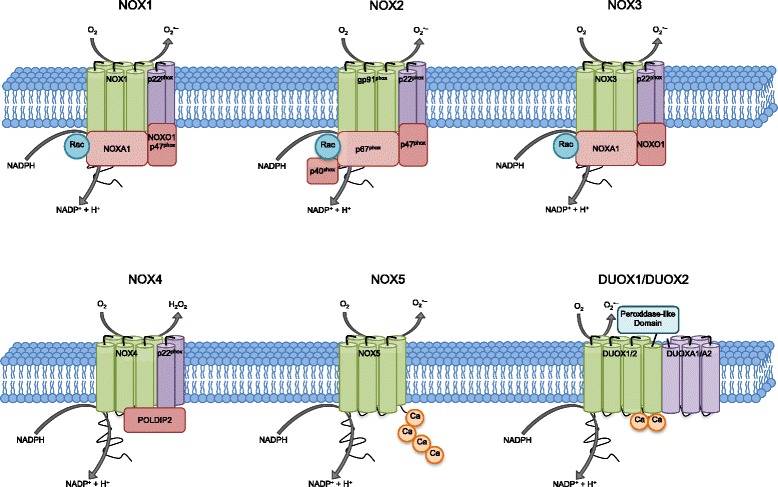
NOX isoforms and regulatory subunits. NADPH oxidases have a primary role to generate ROS. Several components and domains make up the transmembrane active enzyme complexes of NADPH oxidase isoforms. NOX1-5 and DUOX1/DUOX2 are shown here. Upon activation, an electron will be transferred from NADPH to O_2_ to form superoxide. NOX4 generated superoxide undergoes rapid conversion into hydrogen peroxide, which mediates many of its downstream effects. NOX5 and the DUOX enzymes are sensitive to cellular Ca^2+^ concentrations

The NADPH oxidase (NOX) family of enzymes are transmembrane carriers that transport an electron from cytosolic NADPH to reduce oxygen to superoxide anion [12]. To date, there are seven known members which combine with various subunits to form active enzyme complexes - NOX1, NOX2, NOX3, NOX4, NOX5, dual oxidase 1 (DUOX1), and DUOX2 [34–37] (Fig. 2). The NOX and DUOX isoforms are structurally similar with each isoform having binding sites for heme, flavin adenine dinucleotide (FAD) and NADPH [38, 39], and 6 conserved transmembrane α-helices [12]. DUOX enzymes have an additional seventh transmembrane helix and possess peroxidase activity [12]. Once the active complex is assembled, an electron from cytosolic NADPH is transferred to oxygen on the extracellular side [12]. The distribution and regulation of the NOX and DUOX isoforms throughout the body and brain varies depending upon the isoform. Table 1 summarizes the reported distribution of NOX and DUOX isoforms throughout the body, including the central nervous system (CNS). It also lists the major corresponding regulators and products for the various isoforms.

**Table 1:** Tissue and cellular distribution of NOX isoforms

ISOFORM	Regulators	Product	Reported Non-CNS Distribution	CNS tissue
NOX1	NOXO1 NOXA1 Rac1 PKA PKC MAPK Hsp90	Superoxide	Colon [35, 39, 42] Blood vessels [70, 359, 360] Heart [361] Bone marrow [362] Cochlea [103]	Cerebral cortex [53, 139] Hippocampus [50, 202] Cerebellum [363] Substantia nigra [364] Striatum [61] Hypothalamus [361] Cerebral vessels [359, 365]
NOX2	P67phox P47phox P40phox Rac Hsp90	Superoxide	Vasculature [365, 366] Phagocytes [319, 367] Heart [361, 368, 369] Kidney [370] Gastrointestinal tract [371] Liver [372] Pancreas [373] Cochlea [103]	Cerebral cortex [71, 374, 375] Hippocampus [132, 374, 376] Cerebellum [363] Striatum [377, 378] Substantia nigra [379, 380] Hypothalamus [361, 381] Subfornical organ [382] Lateral ventricle [383] Subventricular zone [383] Midbrain [380] Nucleus tractus solitarius [384]
NOX3	NOXA1 NOXO1 Rac p67phox	Superoxide	Inner ear/Cochlea [100, 102, 103, 385] Lungs [386, 387]	Cerebral cortex [71]
NOX4	p22^phox^ PolDip2 PDI Hypoxia mGlu3 PI3K TLR4	Hydrogen peroxide	Ubiquitous, including: Kidney [35, 39, 388] Vessels [389, 390] Lung [391–394] Bone [39, 362, 395] Placenta [35, 39, 396] Cochlea [103]	Cerebral cortex [71, 377, 397] Hippocampus [376] Cerebellum [363] Hypothalamus [361, 381, 398] Subfornical organ [382] Glioblastoma [35]
NOX5	Ca^+2^ Hsp90	Superoxide	Testis [35] Ovary [35] Spleen [35] Kidney [399] Vasculature [400, 401] Uterus [402] Cochlea [103] Tumors [35, 115]	Glioblastoma [35, 116]
DUOX1	Ca^2+^ Duoxa1 Duoxa2	Hydrogen peroxide	Thyroid [34, 37] Lung [354, 403, 404] Skin [154, 156, 405] Cochlea [103]	None reported
DUOX2	Ca^2+^ TRIF/NFkB MyD88/p38 Duoxa1 Duoxa2	Hydrogen peroxide	Cochlea [103] Thyroid [34, 37] Lung [125, 354] GI tract [152, 403, 406] Salivary gland [403]	None reported

#### NOX1

NOX1 generates the product superoxide, and has the most similarity to the NOX2 and NOX3 isoforms, as evidenced by association with homologous regulatory subunits and regulation by Rac GTPase [40–42]. As shown in Fig. 2, NOX1-3 subunits form functional dimers with p22phox. In addition, NADPH oxidase activator 1 (NOXA1) serves as an “activator” for NOX 1 and 3 [43], and is structurally homologous to p67 phagocytic oxidase (p67phox), which is the activator for NOX2. NADPH oxidase organizer 1 (NOXO1) is an “organizer” for NOX1 and 3 [43, 44], and is structurally homologous to p47phox and p40phox, which are organizers for NOX2. Superoxide anion production by NOX1 has been shown to require phosphorylation of NOXO1 by protein kinase A (PKA) (at Ser154) or PKC (at Thr341), which then interacts with NOXA1 to regulate NOX1 activity [45]. However, it is important to note that NOXO1 is localized at the membrane independent of cellular activation, which combined with its lack of auto-inhibitory function, may contribute to constitutively activated NOX1 [44]. Additional work has shown that phosphorylation of NOXA1 on serine 282 by MAP kinases and on serine 172 by PKC down-regulates constitutive NOX1 activity [46]. In addition, phosphorylation of NOX1 (at Thr429) by PKC-β has been reported to be critical for NOX1 complex formation with the cytosolic regulatory units and thus for NOX1 activation [47, 48]. Furthermore, as shown in Fig. 2, Rac1 is also a known activator of NOX1 [41]. Finally, a number of enzymes, cytokines, growth factors, hormones, and cell stressors have been implicated to also regulate NOX1 [49–60]. Functionally, NOX1 has been implicated in pain that accompanies inflammation, host defense via expression in microglia, and neuronal growth [61–64]. A role for NOX1 in neuropathology and neurodegenerative disorders has also been implicated and will be discussed in subsequent sections below.

#### NOX2

NOX2 (sometimes referred to by its major subunit name - gp91phox) generates superoxide as its product [12, 65, 66]. NOX2 is expressed in various tissues of the body as well as several regions of the brain (see Table 1). At the cellular level, NOX2 has been reported to be expressed in various cell types, including neurons and endothelial cells [67–69], and is heavily expressed in microglia, where it is involved in immune/inflammatory responses [70], particularly after injury [71, 72]. As shown in Fig. 2, NOX2 has both membrane subunits (gp91phox and p22phox) and cytosolic components (p47phox and p67phox). During activation, a phosphorylated p47phox interacts with p22phox, which facilitates translocation to the membrane where p67phox can bind to p47phox to form the active NOX2 complex [73–75]. Rac2 is coordinately recruited to the membrane, where it binds directly to p67phox and is essential for NOX2 activation [76, 77]. Binding sites for Rac2 have also been identified directly in NOX2 [78]. NOX2 in non-phagocytic cells is structurally similar to NOX2 of phagocytes, but its regulation may differ. A resting endothelial cell has partially preassembled NOX2 that generates low levels of superoxide intracellularly for physiological functions rather than having functional activity bound to the plasma membrane such as in neutrophils [79]. Activation of NOX2 requires serine phosphorylation of the p47phox subunit in models using TNF stimulation [80, 81] and angiotensin II stimulation [82]. Furthermore, a number of enzymes, cytokines, metabolic factors, and cell stressors (hypoxia and Aβ) have been implicated to regulate NOX2 [53, 68, 83–89]. Identified downstream targets of NOX2-derived ROS include various kinase signaling pathways [90–95], as well as cytokines and transcription factors [80, 96–99].

#### NOX3

In the brain, NOX3 has been reported to be expressed in neurons in the inner ear [71], where it has been implicated to play a significant role in the cochlea and vestibular system [12, 100, 101]. In support of this contention, NOX3 knockout mice have been reported to show signs of imbalance [102]. In addition, reduction of NOX3 has been implicated to have a protective effect in cochlear injury by reducing the amount of oxidative stress produced [103]. NOX3 generates superoxide as its product, and the regulatory mechanism of NOX3 can involve Rac1, NOXO1, and p67phox [40, 64, 104]. Furthermore, it has been suggested that the p22phox subunit is critical for the proper assembly and function of NOX3 [105, 106] (see Fig. 2 and Table 1). Finally, NOX3 has also been reported to be active in absence of its known regulators [104].

#### NOX4

NOX4 is a constitutively activated isoform [107, 108], whose expression however is inducible. Unlike the other NOX isoforms, the downstream effects of NOX4 are thought to be mediated by hydrogen peroxide, due to the rapid conversion of NOX4-generated superoxide to hydrogen peroxide [109]. As shown in Table 1, NOX4 is widely expressed in the body, and is expressed in various brain regions and cells. It is reported to be a major source of superoxide in human pericytes [110]. NOX4 requires the p22phox subunit for function [108] and has been suggested to be inducible due to the close association between NOX4 mRNA and ROS generation [109]. In addition, other NOX4 regulatory proteins have been identified (Table 1), such as polymerase (DNA-directed) delta interacting protein 2 (Poldip2) [111], protein disulfide isomerase (PDI) [112], and tyrosine kinase substrate with 4/5 SH3 domains (Tsks4/5) [113], which may further regulate the activity of NOX4. In addition, recent evidence suggests that hydrogen peroxide-inducible clone 5 (Hic-5), a focal adhesion adaptor protein, functions as a negative regulator of NOX4 by promoting the ubiquitin-proteosomal system-mediated degradation of NOX4 [114].

#### NOX5

First reported in 2001, NOX5 is genetically the most distinct of the NOX isoforms [35, 115]. As shown in Table 1, NOX5 is highly expressed in the spleen and testis, and to a lesser extent in other tissues of the body. Expression of NOX5 in the brain has only been documented in brain cancer, specifically glioblastoma multiforme [116]. As shown in Fig. 2, NOX5 is distinct from the other NOX isoforms in that it forms fully functional oligomers [117]. Finally, phosphorylation of NOX5 on Thr494 and Ser498 residue has been reported to increase its sensitivity to calcium [118].

#### DUOX1/2

Dual oxidases one and two (DUOX1/2) generate hydrogen peroxide, and are named for their gp91phox domain and their extracellular peroxidase domain. DUOX1 and DUOX2 have been most studied in the thyroid [34, 37], but have also been found in other parts of the body (see Table 1). As shown in Fig. 2, DUOX enzymes interact with DUOX activator proteins, DUOXA1 and DUOXA2, which are critical for targeting and function of the DUOX enzymes [119]. DUOX2 is reported to be involved in thyroid hormone synthesis, and missense mutation of DUOX2 results in congenital hypothyroidism [120]. DUOX1 is also expressed in the thyroid gland, but its function in the thyroid is unknown. Recently, investigations on the DUOX enzymes have expanded to other body systems. DUOX1 has been reported to mediate allergic asthma [121], prolong ROS generation after irradiation [122], and play a role in cancer development [123]. DUOX2 has been reported to mediate keratinocyte inflammation [124], cochlear injury [103], and lung injury [125]. Furthermore, both DUOX enzymes have been implicated to be important in ROS-dependent immunity [126].

### Evidence of a role of NADPH oxidase in brain injury and neurodegenerative disorders

There is growing evidence of a role of NOX isoforms in brain injury and neurodegenerative disorders, particularly for NOX1, 2, and 4. NOX activation can be increased by a variety of inflammatory and neurodegenerative factors, such as amyloid precursor protein (APP), amyloid β, tumor necrosis factor-alpha (TNF-α), matrix metalloproteinase (MMP), interleukins, and α-synucleins, as well as neuronal damage and cell death [75, 80, 127, 128]. Evidence supporting a role of NOX activation in the pathology of ischemic and traumatic brain injury, as well as several major neurodegenerative disorders is discussed in detail below. Table 2 summarizes animal studies showing effects of genetic deletion or knockdown of NOX in the following disease models.

**Table 2:** Animal studies using genetic manipulations of NADPH oxidases on neurodegenerative disorders

Disorder	Animal model	Genetic manipulation	Manipulation Result	References
Alzheimer’s Disease	Aβ_1–42_ injection	p47^phox^ −/−	↑M2 microglial phenotype (Ym1) ↓Monocyte chemoattractant (CCL2)	[205]
	Tg2576	Nox2 −/−	↓Cerebrovascular dysfunction & ROS ↑Cognition	[407]
	Tg2576 Aβ_1–40_ injection	Nox2 −/−	↓ROS & vascular dysfunction	[204]
	APP-expressing neuroblastoma	Nox2 −/− macrophages (PLB-985 X-CGD)	Macrophages unable to kill APP-expressing neuroblastoma cells	[408]
Stroke	tMCAO	Nox1 −/−	↑Cortical (but not total) infarct	[59]
	tMCAO	Nox1 −/−	↓Lesion, edema & BBB disruption	[409]
	pMCAO	Nox1 −/−	No change in infarct size	[409]
	tMCAO	Nox1 −/−	No change in infarct size or outcome	[140]
	tMCAO	Nox2 −/−	No change in infarct size or outcome	[140]
	tMCAO	Nox4 −/−	↓Infarct, edema & BBB disruption ↑Cognition/basal motor function	[140]
	pMCAO	Nox4 −/−	↓Infarct & functional deficits	[140]
	tMCAO	Nox2 −/−	↓Infarct	[160]
	tMCAO	Nox2 −/−	↓Lesion & BBB disruption	[158]
	Perinatal Hypoxia-Ischemia (HI)	Nox2 −/−	No change in infarct size (severe hypoxia); ↑Infarct (moderate ischemia)	[410]
	tMCAO	Nox2 −/−	↓Infarct & spectrin cleavage ↑Neurological outcome	[131]
	tMCAO	Nox2 −/−	↓Infarct & inflammation	[157]
	tBCCAO SAH	p47^phox^ −/− Nox2 −/−	↓CA1 neuronal degeneration No change in mortality, brain water content, or intensity of oxidative stress	[411] [172]
	ICH	Nox2 −/−	↓Hematoma, edema, deficits, mortality	[171]
Traumatic Brain Injury	CCI	Nox2 −/−	↓Lesion, apoptosis & ROS	[185]
	SBI	Nox2 −/−	↑Neurological outcome	[412]
Parkinson’s Disease	6-OHDA	Nox2 −/−	↑Neurological outcome & resistance to neurotoxicity	[227]
	MPTP	Nox2 −/−	Neuroprotective	[218]
	MPP^+^	Nox2 −/− neuron-glia culture	↓Dopaminergic neurodegeneration	[221]
	6-OHDA	Nox2 −/−	↓Rotational behavior	[217]
	Paraquat	NOX1 −/− differentiated human dopaminergic cells	↓α-synuclein expression & aggregation	[226]
	Paraquat	Nox1 shRNA	↓α-synuclein expression & aggregation, ROS, dopaminergic neuronal loss	[226]
Amyotrophic Lateral Sclerosis	SOD1^G93A^	Nox2 −/−	↓ROS, protein carbonylation, neurodegeneration ↑Longevity	[262]
	SOD1^G93A^	Nox1 −/−	Delayed disease progression ↑Survival	[276]
	SOD1^G93A^	Nox2 −/−	Delayed disease progression ↓Muscle atrophy ↑Survival	[276]
	SOD1^G93A^	Nox1 +/− or Nox2 +/−	↑Survival	[276]
Huntington’s Disease	HD^140Q/140Q^	Nox2 −/−	↑Neuronal viability ↓ROS	[246]
Multiple Sclerosis	MOG induced EAE	p47^phox^ −/− BV-2-myelin culture	↓ROS, neuroinflammation	[308]
	MOG induced EAE	Nox2 −/−	↓Weight loss, microglial reactivity ↑Oligodendrocyte survival, neurological outcome	[311]
	MOG induced EAE	p47^phox^ −/−	↓EAE presentation	[309]
	MOG induced EAE	p47^phox^ splice mutation	Enhanced EAE presentation	[310]

#### Stroke

##### NOX activation in stroke

Tissue injury in stroke is caused paradoxically by both the depletion of oxygen (ischemia) and the replenishment of oxygen (reperfusion). Increased levels of ROS have been proposed to have clinical significance in both stages of stroke injury [129, 130]. Furthermore, there is considerable evidence that NOX-mediated ROS is a major mechanism of neuronal damage in stroke pathology [12, 131–133]. To date, NOX2 and NOX4 are the isoforms most implicated to contribute to ROS generation and pathology following cerebral ischemia, although there is also some evidence for NOX1 involvement as well. Both focal and global cerebral ischemia models have been used in the study of NOX activation. Using a transient global cerebral ischemia (tGCI) model, our group reported a 6–7 fold elevation of NOX2 (gp91phox) membrane complex formation, NADPH oxidase activity, and superoxide production in the hippocampus after tGCI [132]. Additional studies revealed that NOX2 is primarily localized in neurons and endothelial cells at early time-points (3–6 h) after tGCI, followed by expression in microglial cells at later time-points (72 hr) [132, 134]. Likewise, studies in focal cerebral ischemia animal models also found that NOX2 and NOX4 mRNA and protein, as well as NOX activity are elevated in the peri-infarct region of the cerebral cortex from 2–48 h after reperfusion [135–138]. Few studies have examined later time-points, but one study found NOX2 protein expression was markedly elevated in the peri-infarct region of the cortex up to 2 weeks after focal cerebral ischemia [139]. Similar to the finding in rodent models, there is also a report that NOX4 immunostaining is increased in neurons and vascular endothelial cells in the cerebral cortex of human patients after stroke, although the patient sample size in the study was very small [140]. As was the case in the tGCI model, expression of NOX2 and NOX4 was noted in *multiple* cell types after focal cerebral ischemia, including neurons, astrocytes and microglia. Interestingly, superoxide production and NOX2 expression was also shown to be significantly elevated in mouse cerebral arteries at 24 h after focal cerebral ischemia, and suggested to contribute to cerebral ischemia-induced vascular dysfunction [141]. Finally, there is a single report that NOX1 protein expression is significantly increased in the peri-infarct region of the cortex at 1d, 1 week, and 2 weeks after focal cerebral ischemia, although this remains to be confirmed [139].

An increase in NOX activation has also been reported in studies utilizing hemorrhagic models. Enhanced immunoreactivity to gp91phox associated with neuronal injury and increased gp91phox mRNA expression was reported in the rat cortex at 24 h after subarachnoid hemorrhage (SAH) [142, 143]. Another study reported an elevation of superoxide beginning as early as 12 h after SAH [144]. Elevated NOX activity and superoxide production were associated with increased membrane translocation of the p47phox subunit [145] and increased membrane translocation of a NOX activator Rac [144] at 24 h after SAH. The activation of NOX in SAH appears to be short lived as one study found NOX activity return to baseline by 48 h after SAH, with no changes in expression of gp91phox or p22phox throughout the experiment [145]. A similar acute NOX activation pattern was seen in hemorrhagic stroke where NOX2 protein levels were shown to be increased in the striatum at 12 h, and peaking at 24 h after intracerebral hemorrhage (ICH) [146]. Gp91phox was primarily expressed in activated microglia and colocalized with peroxynitrite at 24 h after ICH in the injured hemisphere [147]. Human data for NOX activation in hemorrhagic stroke models are scarce, but a study of the Chinese Han population resulted in the identification of a p22phox polymorphism associated with increased NOX activation and susceptibility to ICH [148]. While clearly more studies using different injury models are needed to solidify the evidence for NOX involvement in the pathogenesis of hemorrhagic stroke, the current findings suggests that NOX activation is involved in the acute phase of hemorrhagic stroke, as opposed to a more prolonged involvement in ischemic stroke.

##### Neuroprotective effect of NOX inhibition in stroke

In agreement with the induction of NOX isoforms and elevated NADPH oxidase activity after cerebral ischemia, NOX inhibitor and knockout studies support a critical role for NADPH oxidase in ROS generation, oxidative stress damage, inflammation, and functional outcome following cerebral ischemia (Table 2). For instance, a NOX2 selective inhibitor, gp91ds-tat, but not a scrambled control peptide, was shown to significantly attenuate the increase in NADPH oxidase activity and superoxide level in the hippocampal CA1 region after tGCI, and was strongly neuroprotective when administered 30 mins before induction of GCI [132]. Further work showed that gp91ds-tat reduced acetylation and activation of p53 and reduced expression of the apoptotic factor, puma in the rat hippocampus after tGCI [149]. Many cerebral ischemia studies have used apocynin, a non-isoform-specific NOX inhibitor. It should be noted that apocynin has also been reported to have direct anti-ROS scavenging ability [150]. Nevertheless, apocynin has been shown to reduce superoxide elevation, oxidative stress, post-ischemic inflammation, and infarct volume, and improve neurological function in focal cerebral ischemia animal models [131, 151–156]. These studies used a dose range from 2.5-50 mg/kg and either an intravenous or intraperitoneal administration route in mice and rats. Apocynin treatment was initiated at various times ranging from −60 min to +120 min relative to the initiation of stroke. This indicates that apocynin can be protective at least out to 2 h after the onset of stroke, although it should be pointed out that some dissenting studies failed to find post-stroke protection with apocynin [140, 151], possibly due to missing a time window of therapeutic apocynin application or the narrow therapeutic dose range of apocynin [155]. Interestingly, the ability of apocynin to reduce infarct size after focal cerebral ischemia is lost in NOX2 knockout mice, suggesting its effects require NOX2 [151].

Knockout animal studies have further implicated NOX2 and NOX4 as the principal NOX isoforms involved in mediating oxidative stress and brain injury following cerebral ischemia (Table 2). For instance, a majority of studies have reported that NOX2 knockout animals have significantly reduced infarct size following focal cerebral ischemia [131, 153, 157–160], although there are a few dissenting studies [140, 161]. NOX2 knockout animals also have been demonstrated to have reduced ROS and oxidative stress in the brain after focal cerebral ischemia [131, 159], as well as reduced blood brain barrier disruption [158], and significantly attenuated post-ischemic inflammation [131, 157].

Mechanistically, studies have shown that glutamate excitotoxicity plays an important role in brain damage following cerebral ischemia [162–164]. Recent work suggests a role for NOX2 in regulating post-stroke glutamate excitotoxicity. For instance, NOX2 has been demonstrated to mediate expression of complexin II, which via SNARE signaling induces glutamate excitotoxicity [165]. Similarly, NOX activation appears necessary for the NMDA path of excitotoxicity. The active transmembrane complex composed of p40phox, p22phox, and gp91phox produces superoxide extracellularly and H^+^ intracellularly, where a Na^+^/H^+^ exchanger removes intracellular H^+^ to maintain NOX activity. When the Na^+^/H^+^ exchanger is inhibited, NOX activity appears to decrease as shown by decreased superoxide production and attenuated excitotoxicity [84]. These results highlight the role of NADPH oxidase in mediating NMDA excitotoxicity.

Furthermore, there is evidence that NADPH oxidase is critical for the release of neurotoxic factors from activated microglia after ischemia. For instance, chemical ischemia has been shown to induce NOX2 elevation and ROS generation in cultured BV2 microglia cells, and conditioned media from the activated microglia induces robust neuronal cell death in vitro [166]. Moreover, NOX2 siRNA knockdown or treatment with the NOX inhibitor, apocynin blocked the cell death-inducing effect of the activated microglia-conditioned media [166]. An important question is what is the contribution of NOX activation in resident microglia in the brain versus NOX activation in circulating immune cells. Tang et. al. attempted to address this question by performing bone marrow chimera studies using wild-type and NOX2-KO mice [153]. The study revealed that wild-type mice receiving NOX2-KO bone marrow had smaller infarct sizes and less hemorrhage than NOX2 KO mice receiving wild-type bone marrow. The authors concluded that while NOX2 from both resident brain cells and circulating immune cells contributed to ischemic brain injury and hemorrhage, based on the bone marrow chimera results, NOX2 from the circulating cells might contribute more to exacerbation of brain ischemia than brain resident cells.

Finally, in addition to the role for NOX2 in mediating cerebral ischemia brain damage, there is one report that showed NOX4 knockout mice have reduced oxidative stress and neurodegeneration following focal cerebral ischemia [140]. Likewise, administration of the NOX inhibitor VAS2870 at 2 and 12 h after tMCAO also reduces ischemic brain injury after focal cerebral ischemia, an effect possibly due to inhibition of NOX4 [140]. Moreover, mice engineered to overexpress NOX4 in brain pericytes had significantly greater infarct volume after focal cerebral ischemia, along with enhanced reactive oxygen species production and blood–brain barrier breakdown in the peri-infarct region [167]. VAS2870 IV treatment in a tMCAO model showed increased expression of microRNAs targeting NOX2 and NOX4, suggesting that the neuroprotective effects of VAS2870 may also be mediated through regulation of microRNAs [168]. Taken together, the above studies indicate that NOX2 and NOX4 play an important role in infarct damage, inflammation and neurological deficits that occur following cerebral ischemia.

The evidence of NOX inhibition in hemorrhagic stroke is more limited. A study of SAH using diphenyleneiodonium chloride (DPI) to inhibit NOX ameliorated the decreased MCA luminal diameter seen after SAH [145], indicating a potential for NOX inhibition to reduce cerebral vasospasms that may complicate SAH. In studies investigating hemorrhagic transformation after ischemic stroke, NOX inhibition via VAS2870 was able to reduce infarct volume and attenuate NOX2 and NOX4 expression in rats, which correlated with better outcome and reduced hemorrhagic transformation after reperfusion in acute ischemic stroke [169]. Another study found that apocynin treatment at high doses did not improve outcome in a collagenase model of rat ICH [170], although the narrow neuroprotective dose range of apocynin may account for the lack of neuroprotection seen at high doses [155]. Modulation of NOX expression or activity has also been implicated in the protective effects of hyperbaric oxygen in SAH [142, 143], Rac inhibition in SAH [144], adiponectin paralog in ICH [146], and Brilliant Blue G in ICH [147]. As these treatments are not specific in targeting NOX, the value of NOX as a therapeutic target in hemorrhagic stroke remains to be validated with more studies utilizing NOX-specific inhibitors. A few knockout studies have been done in investigating the role of NOX enzymes in hemorrhagic stroke. Gp91phox KO mice show decreased oxidative products, reduced brain edema, smaller hematoma size, decreased neurological deficits, and decreased mortality as opposed to wild type mice after collagenase induced ICH [171]. However, an endovascular perforation model of SAH with gp91phox KO mice showed no difference in the cerebral blood flow, brain edema, intensity of oxidative stress, or mortality between WT and KO mice [172]. It is uncertain whether different disease models or the acute window of NOX activation in hemorrhagic stoke may account for the variability in reported data. These preliminary studies with NOX KO mice in hemorrhagic stroke require further validation.

#### Traumatic brain injury

##### NOX activation in traumatic train injury

In addition to a role in stroke, there is growing evidence that NOX activation contributes significantly to the neuropathology of traumatic brain injury (TBI). TBI has a nearly 40% mortality rate [173] and is the leading cause of death in young people, especially with increasing rates of motor vehicle accidents [174]. In recent years, TBI has gained recognition as a chronic and progressive disorder resulting from an acute trauma [175]. The primary mechanical injury over time leads to development of the secondary injury such as edema, ischemia, hypoxia, neuroinflammation, and neurodegeneration of the cortex and underlying hippocampus [176–178]. Oxidative stress is thought to play a key role in the development of cerebral edema, breakdown of the blood brain barrier (BBB), and the secondary neuronal damage found post-TBI. The initial focal injury spreads via inflammatory signals to previously healthy regions nearby and amplifies the original tissue injury [179, 180]. BBB disruptions allow increased permeability that contribute to the cerebral edema following TBI [181–183].

Since oxidative stress plays a key role in TBI, several groups have examined the role of NOX isoforms, particularly NOX2 activation in TBI pathology. Studies from our group and others showed that NOX2 expression and NADPH oxidase activity increases rapidly in the mouse cerebral cortex and hippocampal CA1 region in an animal model of TBI (controlled cortical impact), with an early peak at 1 h, followed by a secondary peak from 24–96 h after TBI [133, 184]. The first early peak of NADPH oxidase activity was in neurons, while the later second peak primarily occurred in microglia [133, 184, 185]. Further work showed that NOX2 expression remained elevated above sham controls at 7 days and 28 days post-TBI in rats [71]. At these later stages, pro-inflammatory M1 microglia were reported to have high NOX2 expression after TBI in a controlled cortical impact model in mice [186]. In fact, NOX2 may contribute to persistent microglia activation that has been observed in the cortex chronically after TBI, as NOX2 was found to be expressed in highly activated microglia at the margins of the expanding lesion at 1-year after the TBI [175]. NOX4 has also been reported to be elevated in the cortex after controlled cortical impact or repeated blast injury in rats [71, 187]. NOX3 was shown to be present in neurons constitutively in both injured and uninjured animals, and its role in TBI remains unclear [71]. Other NOX isoforms have not been investigated in the context of TBI.

Similar to the results obtained in animal studies described above, immunofluorescent staining of human post-mortem TBI brain samples showed that NOX2 and NOX4 expression was increased in the cortex at 6–24 and 24–48 h after TBI, respectively, and abnormal NOX2 and NOX4 expression showed correlation to patient prognosis [188]. NOX4 expression has also been reported to be increased in postmortem brain samples from athletes diagnosed with chronic traumatic encephalopathy, and the increase in NOX4 activity correlated with increased superoxide formation [187]. Increased ROS production and elevated expression of NOX2 has also been reported in circulating neutrophils after TBI in humans, which paralleled a significant increase in plasma levels of inflammatory cytokines [189]. These findings suggest an increased systemic inflammatory response occurs after TBI, which may involve facilitation by NOX2. The systemic inflammatory response after TBI could cause increased local secondary damage in the brain, as well as systemic damage to bystander peripheral tissues. Targeting NOX inhibition in these systemic neutrophils could potentially protect uninjured organs from secondary damage following TBI.

##### Neuroprotective effect of NOX inhibition in traumatic brain injury

To examine a potential *causative* role of NADPH oxidase in TBI pathology and outcome, investigators have used NADPH oxidase inhibitors and NOX2 knockout animals. Administration of the NADPH oxidase inhibitor, apocynin markedly inhibited pro-inflammatory microglial activation and oxidative stress damage to neurons [71, 133, 184, 190], as well as attenuated TBI-induction of AD-related proteins and axonal damage markers, such as β-amyloid and amyloid precursor protein [133]. Furthermore administration of apocynin or the more specific NOX2 inhibitor, gp91ds-tat, induced significant neuroprotection against TBI and reduced neurological deficits [133, 184, 191]. Administration of gp91ds-tat also altered microglia polarization after TBI to favor the M2-like anti-inflammatory phenotype [186], suggesting an important role of NADPH oxidase in promoting the “pro-inflammatory” M1-like microglia phenotype after TBI. Similarly, NOX2 knockout mice have decreased contusion size, as well as decreased superoxide and peroxynitrite metabolism, and a decreased number of apoptotic cells in the cortex after TBI, confirming a possible role for NOX2 in TBI pathology [185] (Table 2). While NOX4 has been reported to be elevated after TBI (like NOX2) [71, 187, 188], there have been as yet no comparable studies examining TBI pathology or outcome in NOX4 knockout mice. Thus, studies to address this deficit are needed.

#### Alzheimer’s disease

##### NOX activation in Alzheimer’s disease

Alzheimer’s disease (AD) is characterized by progressive memory loss, cognitive and executive impairment, with varying degrees of aphasia, apraxia, and agnosia [98, 192]. Early in AD progression, cognitive symptoms are caused by damage to the hippocampus [193, 194]. Oxidative stress, particularly that created by NOX2 induction, has been implicated to have a significant role in the development of AD [24, 195, 196]. Postmortem analyses of cerebral cortices of human AD patients showed increased p47phox and p67phox expression and translocation to the membrane in AD brains [196], suggesting that NOX is activated in AD. Antemortem behavioral testing and postmortem histopathological assessment (of frontal and temporal cortex) in patients with either no cognitive impairment, preclinical AD, mild cognitive impairment, early AD, or mild-to-moderate AD, confirmed elevation of p47phox, p67phox, p40phox and p22phox, as well as elevation of NOX activity in mild cognitive impairment and AD patients [197]. There was also a robust correlation between NOX activity and an individual's cognitive status (e.g. as NOX enzyme activity increased, cognitive performance decreased). The enhanced induction of NOX isoforms and activity in AD, may be due, in part, to elevated β-amyloid (Aβ) levels, as treatment of microglia with Aβ has been shown to induce p47phox and p67phox subunit translocation to the cell membrane, indicating an activation of microglial NOX and ROS generation [196]. Furthermore, increased expression of NOX2 and oxidative stress has been reported in activated microglia surrounding Aβ-laden capillaries from patients with cerebral amyloid angiopathy [198, 199].

In addition to elevation of NOX2, there is evidence that NOX4 expression is significantly increased in the brain of aged humanized *APPxPS1* knock-in mice, and that there is a significant linear correlation between NOX activity and age-dependent increases of Aβ (1–42) and cognitive dysfunction [200]. Another study showed that NOX1 and NOX3 mRNA levels are increased in the brain during early stages of AD [201]. Interestingly, NOX1 mRNA levels were also reported to be significantly elevated in the hippocampal CA1 region in a bilateral cerebral artery occlusion (BCCAO) rodent model of vascular dementia, and the effect correlated with increased superoxide and ROS levels [202]. As a whole, these studies provide correlational evidence of increased NADPH oxidase subunit expression and NADPH oxidase activity in the brain in AD, which could contribute to AD pathology. Below we discuss evidence from inhibitor and knockout/knockdown studies that support a role for NADPH oxidase in AD pathology and progression.

##### Neuroprotective effect of NOX inhibition in Alzheimer’s disease

In support of NOX2 elevation having an important pathological role in AD, treatment with the NADPH oxidase inhibitor, apocynin markedly reduced oxidative stress, cerebral amyloid angiopathy, and cerebral amyloid angiopathy-induced vasomotor impairment in an animal model of AD (aged APP-overexpressing Tg2576 mice) [203]. As shown in Table 2, other studies showed that NOX2 inactivation by use of NOX2 knockout mice crossed with Tg2576 mice or use of the specific NOX2 inhibitor, gp91ds-tat also significantly reduced oxidative stress, improved the neurovascular dysfunction, and ameliorated behavioral deficits in aged Tg2576 mice [204]. Surprisingly, the beneficial effect of NOX2 inactivation in aged TG2576 mice occurred without reductions in brain amyloid-beta peptide levels or amyloid plaques. However, treatment with the NOX2 inhibitor, gp91ds-tat or knockout of NOX2 has been shown to significantly attenuate the ability of Aβ to induce ROS in the brain [204], suggesting that NOX2 is a major source of ROS induced by Aβ. Furthermore, examination of human AD postmortem brain samples found that microglia “proinflammatory” (M1) marker gene expression is increased, while expression of the anti-inflammatory (M2) marker genes were decreased, as compared to age-matched controls [205]. This effect was correlated with an increase of the NOX2 p47phox subunit in the AD brain, and further studies revealed that p47phox KO mice and apocynin-treated mice showed a switch from “proinflammatory” M1 to the “anti-inflammatory” M2 microglia phenotype following induction of neuroinflammation [205]. These results suggest that NADPH oxidase may facilitate induction of the proinflammatory M1 microglia phenotype during neuroinflammation and AD. Finally, while NOX1, NOX3 and NOX4 have been reported to be elevated in AD, their precise role and contribution to AD is currently unclear. Therefore, more studies are needed to assess the potential role of these NOX isoforms in AD.

#### Parkinson’s disease

##### NOX activation in Parkinson’s disease

Parkinson’s disease (PD) is the second most common neurodegenerative disorder following AD, with progressive symptoms such as resting tremor, rigidity, akinesia, and stooped posture caused by a characteristic loss of dopaminergic neurons in the substantia nigra pars compacta [206–208]. Models of PD often use the neurotoxins, 6-hydroxydopamine (6-OHDA), paraquat, 1-methyl-4-phenyl-1,2,3,6-tetrahydropyridine (MPTP) and rotenone, all of which kill dopaminergic neurons via mechanisms that seem to converge on oxidative stress [209]. Human PD brains have shown evidence of oxidative damage in degenerating areas such as the substantia nigra [210]. Post-mortem analysis and animal studies suggest a role of inflammation in PD pathology [211, 212], as evidenced by increased glial activation [213], which appears to be a persistent chronic activation [214]. Microglia have been suggested to contribute to the damage of dopaminergic neurons in PD via NOX-derived ROS [215]. In support of this contention, NOX1 expression has been reported to increase after striatal injection of 6-OHDA and is associated with a simultaneous increase in DNA oxidative stress [216]. Additionally, following 6-OHDA induction, there is an increase in NOX1 and NOX2 gene expression in the mouse striatum, and NOX2 expression in the substantia nigra [217]. In vivo and in vitro studies of PD induced by paraquat have shown increased expression of NOX1 and synucleins [217]. NOX2 expression has also been shown to increase in mouse brains after administration of MPTP [218] and to be elevated in PD human brains [218]. Likewise, NOX2 and NADPH oxidase activity have been reported to be increased in the substantia nigra in an LPS-induced animal model of PD [219]. These findings demonstrate an elevation of NOX1 and NOX2 may contribute to the dopaminergic neuronal loss that occurs in PD. The mechanisms that lead to NOX induction in PD are unclear; however, α-synuclein, a key protein implicated in PD pathology, has been shown to activate NOX2 in microglia via a process that is mediated by the purinergic P2X7 receptor [220]. Likewise, substance P, which is dysregulated and suggested to contribute to PD pathogenesis, has also been shown to activate NOX2 in microglia, an effect critical for substance P-induced dopaminergic neurotoxicity [221]. Other PD-related genes have been reported to affect NOX activation. DJ-1/Park7 can bind to p47phox to alter NOX activation [222, 223], and silencing of DJ-1 has been shown to increase NOX4 expression and ROS production [224]. In addition, NOX has been implicated as a potential mechanism underlying neuronal vulnerability associated with PINK1 dysfunction [225]. While interesting, further studies are needed using PD models in order to elicit whether these PD-related genes have a functional role in mediating downstream NOX activation.

##### Neuroprotective effect of NOX inhibition in Parkinson’s disease

In support of a role of NOX1 in the pathology of Parkinson’s disease, NOX1 KO mice have a significant attenuation of DNA oxidative stress in a 6-OHDA striatal injection PD model (Table 2) [216]. Furthermore, in both in vitro and in vivo paraquat–induced PD models, NOX1 knockdown significantly reduced α-synuclein expression and aggregation, as well as oxidative stress and dopaminergic neuronal cell loss [226]. With respect to NOX2, NOX2 KO mice exhibited less dopaminergic neuronal degeneration than their controls when lesioned by 6-OHDA [227]. Likewise, dopaminergic neurons from NOX2 KO mice were significantly more resistant to rotenone neurotoxicity (an environmental toxin linked to PD) than those from wild-type mice [228]. The enhanced neurotoxicity of rotenone was suggested to be due to release of NOX2-derived superoxide from activated microglia. This suggestion was supported by the fact that addition of microglia prepared from wild-type mice, but not from NOX2 KO mice, to neuron-enriched cultures markedly increased rotenone-induced degeneration of dopaminergic neurons [228]. Further evidence of a role of NOX2 in PD pathogenesis has come from NOX inhibitor studies. For instance, in a leucine-rich repeat kinase 2 transgenic PD mouse model, treatment with diapocynin, an orally active apocynin derivative, relieved early PD symptoms regarding coordination and balance [229]. Oral diapocynin treatment also exerted anti-inflammatory and neuroprotective effects in a MPTP mouse model of PD, where it preserved locomotor function and halted disease progression in a chronic mouse model of PD [230]. Furthermore, oral apocynin administration in a MPPT monkey model of PD increased the number of surviving dopaminergic neurons and relieved PD symptoms and motor function deterioration [231]. Taken together, these studies implicate a role for NOX1 and NOX2 in PD pathogenesis and targeting them may have therapeutic efficacy and should be explored further.

#### Huntington’s disease

##### NOX activation in Huntington’s disease

Huntington’s disease (HD) is a relentlessly progressive and fatal neurodegenerative disorder involving an autosomal-dominant inheritance of trinucleotide (CAG) repeat expansion in the huntingtin gene, which clinically manifests in choreiform movements, psychiatric disturbances, and cognitive impairments [232–236]. HD shows selectivity for atrophy and loss of medium spiny neurons in the striatum, but eventually can lead to atrophy of the cerebral cortex at later stages of the disease [233, 235]. Though not necessarily a direct effect of the mutant huntingtin, oxidative stress has been implicated as an important mechanism of HD pathology as susceptible neurons may have impaired tolerance of ROS [234, 237, 238]. Studies of human HD brain and tissue samples found increased oxidative damage in the caudate [239], parietal cortex [240], and peripheral blood [241, 242] (as measured by 8-hydroxy-2’-deoxyguanosine), in CSF (as measured by isoprostanes) [243], and in serum (as measured by malondialdehyde and 4-hydroxynonenal) [71] indicating a role of oxidative stress in HD pathology. Furthermore, reduced SOD antioxidant activity in the cortex and cerebellum [239] and in erythrocytes [241] also support an impaired antioxidant capacity in HD patients. However, the evidence surrounding the presence of oxidative damage markers in post-mortem brain tissue is not uniform since a report exists showing unaltered levels of oxidative DNA damage, protein carbonylation, and lipid peroxidation in HD brain samples when compared to controls [138]. Nonetheless, animal studies using transgenic models of HD have provided a stronger consensus in the role of ROS in HD pathology. In HD transgenic R6/2 mice that develop a movement disorder similar to HD presentation at 14–16 weeks, the forebrain showed increased signs of DNA oxidation at 12–14 weeks of age [244]. Despite initial increases in SOD function after disease onset, older mice (35 weeks) show diminished protection by SOD [245]. Regarding potential NOX involvement in HD, human HD patient brain lysates showed increased NOX activity in the cortex and striatum [246]. Furthermore, studies using a rat model of intrastriatal quinolinic acid injection for HD showed increased NOX-linked striatal superoxide production that increased with the addition of NOX substrate, NADH [247]. A model utilizing 3-nitroproprionic acid-induced striatal lesions, which mimics many characteristics of HD [3], likewise found increased hydroxyl free radicals in the lesioned striatum [248]. Furthermore, studies using a mouse model of HD found that NOX activity was significantly elevated in cortical and striatal synaptosomes in HD^140Q/140Q^ mice [246], and in primary neurons derived from HD^140Q/140Q^ mice [246]. A PC12 cell line in vitro study of CAG repeat-expanded proteins also showed increased oxidative damage attributable to NOX [14]. The emerging evidence suggests a role for NOX2 in generating oxidative stress in HD pathology; however, cell type- and isoform-specific effects remains unexplored, and thus additional studies are needed to address this issue.

##### Neuroprotective effect of NOX inhibition in Huntington’s disease

Despite the recent exploration of the role of NOX enzymes in the pathogenesis of HD, there is evidence supporting that inhibition of NOX may be beneficial. In a rat intrastriatal quinolinic acid injection model of HD, both pre- and post-lesion apocynin treatment decreased superoxide levels and attenuated behavioral alterations seen in untreated, lesioned animals [247]. Furthermore, both apocynin and DPI were able to suppress superoxide generation after striatal lesion in the presence of NOX substrate, NADH [247]. This supports that NOX is actively participating in the pathology of this model of HD. Apocynin and DPI treatment of primary cortical and striatal neurons derived from HD^140Q/140Q^ transgenic mice reduced NOX enzyme activity to the levels of WT neurons [246], as well as reduced ROS generation, increased viability, and improved morphology of the neurons [246]. These HD^140Q/140Q^ derived neurons also showed increased neuronal viability when treated either with a superoxide quencher, EUK189, or a more selective NOX inhibitor, VAS2870 [246]. Though VAS2870 has been reported as a selective NOX2 inhibitor, other studies have revealed its effect on other NOX isoforms and cannot be used to implicate NOX2 as the key NOX isoform in HD pathology. However, NOX2 involvement can be studied using gp91ds-tat, a more selective inhibitor of NOX2 [249]. In the PC12 cell line, treatment with gp91ds-tat reduced ROS levels, dissolved nuclear aggregates induced by expanded polyglutamine peptides, and prevented the formation of new aggregates [14], suggesting that NOX inhibition may be beneficial. Though investigations on the role of NOX2 in HD is a recent endeavor, results so far seem to suggest a key role of NOX2 involvement in HD pathology. In support of this possibility, cortical neurons derived from NOX2-deleted HD^140Q/140Q^ mice showed reduced NOX activity and increased cell survival as compared to NOX2-WT HD^140Q/140Q^ mice [246]. These results suggest that NOX2 inhibition can be neuroprotective, and that further studies utilizing NOX2 KO mice in HD models are needed to further confirm the role of NOX2, as well as the potential benefits of NOX inhibition on behavioral parameters, cognitive health, and disease outcome. In addition, additional studies testing the role of other NOX isoforms in HD pathology are also needed.

#### Amyotrophic lateral sclerosis

##### NOX activation in amyotrophic lateral sclerosis

Amyotrophic lateral sclerosis (ALS) is characterized by progressive motor neuron degeneration and subsequent gliosis that burdens patients with muscle weakness and disability, eventually leading to fatal respiratory and cardiac deficits within 3–5 years of diagnosis [250, 251]. Despite over 30 years of research using animal models, only a single drug has been approved for the treatment of ALS (riluzole), and its effect on survival is modest [252]. Although the exact mechanism of motor neuron death in ALS is still under investigation, oxidative stress has been implicated in the disease progression [253, 254]. Patients suffering from either sporadic or familial ALS showed increased oxidative stress markers such as protein carbonylation [255], nitration of protein-tyrosine residues [256], lipid peroxidation [257], protein glycoxidation [257], and mRNA oxidation [258] in their spinal cords. Furthermore, similar increases in oxidative stress markers have also been detected in the brain [259] and CSF [260] of ALS patients. Despite an account where nitrotyrosine was not found to be significantly elevated in the CSF of ALS patients [261], there appears to be a consensus that oxidative stress plays a role in ALS pathology. In particular, post-mortem spinal cord samples from patients with sporadic ALS showed a 3-fold increase in NOX2 expression and increased protein carbonyl adducts than in control post-mortem samples [262]. An animal model commonly used to study ALS is the overexpression of mutant superoxide dismutase one (SOD1), since dominant mutations in SOD1 lead to familial ALS in humans [263]. SOD1 overexpression likely leads to motor neuron damage due to the increase in oxidative stress, in part due to NOX activation. In the most widely used mouse model of ALS (SOD1G93A), NOX2 is upregulated in the ALS mice [262]. Furthermore, ALS mice overexpressing SOD1 had enhanced NOX-dependent superoxide production in the brain and spinal cord [264]. Rac1, an important regulator of NOX, was reported to directly interact with SOD1 [264]. Altered Rac1 modulation by mutant SOD1 (SOD1L8Q) has been implicated in the persistence of NOX dependent superoxide production [264], and binding of mutant SOD1 to Rac1 has been suggested to amplify microglial NOX2 production of ROS [265]. Likewise, human astrocytes that overexpress mutated SOD1 (SOD1G37R) up-regulate NOX2 [266]. In addition to evidence of NOX2 elevation in ALS, human whole genome analysis of sporadic ALS patients linked NOX4 to have a possible role in ALS disease progression [267].

##### Neuroprotective effect of NOX inhibition in amyotrophic lateral sclerosis

Since oxidative stress is implicated in the pathogenesis of ALS, antioxidants have been evaluated for their efficacy to limit disease progression. However, clinical trials based on antioxidants in ALS have been unsuccessful. For instance, Vitamin E [268–270], coenzyme Q10 [271, 272], and edaravone [273], despite slowing motor neuron degeneration in mice SOD1G93A model [274], are a few tested antioxidants and free radical scavengers that have shown no major improvements or limited improvement in quality of life in ALS clinical trials. The lack of translational efficacy may be partially due to pharmacological inability to cross the BBB, variability in experimental conditions, or generalized drug mechanisms that overlook the value of targeting a specific oxidative pathway. Based on the potential role of NOX enzymes in ALS pathology, it is reasonable to investigate whether targeting of NOX can be therapeutic. Studies investigating NOX’s role in human ALS patients found that patients that had lower NOX2 activity in their peripheral blood cells showed significant increases in survival [275]. Additionally, ALS mice deficient in NOX2 showed decreased oxidative stress in spinal cords and a modest increase in lifespan (Table 2) [262]. Likewise, ALS mice having either deletion of NOX1 or NOX2 showed increased survival and delayed disease onset, and these effects are greater in the NOX2 deleted ALS mice [276]. Interestingly, female ALS mice heterozygous for either NOX1 or NOX2 also showed significantly increased survival [276]. Apocynin treatment was also shown to improve motor neuron survival when co-cultured with astrocytes carrying a mutant SOD1 (G37R) [266], further supporting that NOX inhibition can have a neuroprotective role in ALS. Pharmacological inhibition of NOX has also shown benefits in animal models of ALS. For example, long-term treatment with apocynin beginning at an early age (2 weeks) prolonged survival by nearly 50% and increased the number of motor neurons in the spinal cord in hemizygous SOD1^G93A^ mice [264], showing that NOX inhibition can delay ALS progression. Apocynin treatment initiated at a later age (80 days) also showed decreased disease progression, although the effect was minor [264]. In contrast to the above study, another study reported little beneficial effect of apocynin or diapocynin on extending the lifespan in the SOD1G93A model [277]. Clearly, further studies are needed to clarify the role of NOX isoforms in ALS. Use of inducible and cell-specific NOX subunit KO models may also add further clarification and avoid confounds of global KO models.

#### Multiple sclerosis

##### NOX activation in multiple sclerosis

Multiple Sclerosis (MS) is a chronic CNS disease of autoimmune origin that leads to demyelination, impaired motor function, and neurodegeneration [7, 278–282]. Inflammation and neurodegeneration are some of the key characteristics of MS [7, 282, 283]. Accumulating evidence suggests that oxidative stress plays a key role in MS pathology [281, 284–287]. Brain samples from human patients affected with MS show increased signs of oxidative damage to DNA, proteins, and lipids within and outside of MS plaques in the brain [288–294]. It has been suggested that NOX in activated microglia contributes to the production detrimental ROS and plays an important role in MS pathogenesis [295]. NOX2, NOX1, and NOXO1 were shown to be upregulated in activated microglia found in active demyelinating and chronic MS lesions sampled from human MS patient brain tissue [296]. Furthermore, NOX2 was also upregulated in activated microglia in pre-active MS lesions found throughout normal appearing white matter of MS patients [297], suggesting a role of activated microglia in the production of ROS in MS. However, a protective role of ROS has been suggested as decreased ROS production by isolated peripheral blood leukocytes from MS patients were associated with a more severe MS presentation [298]. This suggests that the role of microglia and NOX-mediated ROS production may be more complicated and would need further animal model studies to elucidate the role of NOX in MS pathology. In animal studies, experimental autoimmune encephalomyelitis (EAE) is the most commonly used model to simulate pathological and clinical findings of MS [299]. However, it has been reported that notable differences in observed tissue injury and oxidative stress exist between human MS and animal EAE models where EAE models potentially underrepresent the extent of injury found in human MS studies [300]. Thus, the NOX activation seen in the EAE studies may actually be greater in the human MS model. In vitro, NOX has been shown to increase BBB permeability of brain endothelial cells in EAE [301]. In EAE induced mice, the brain and spinal cord exhibit increased NOX enzyme activity and NOX expression as compared to that of control mice [302, 303], suggesting that NOX activity may contribute to tissue damage in the course of EAE. In particular, NOX2 and the subunits p67, p47, p40, and p22phox mRNA showed increase in both relapsing-remitting and chronic models of EAE [304], indicating a probably increase in oxidative stress during the peak stages of EAE and in the later progressive stage of EAE. In a different study, NOX2 expression was also seen to be elevated in microglia during remission phases of relapsing-remitting EAE [305], which coincided with observed impairments in hippocampal CA1 synaptic plasticity [305]. Despite possible differences between EAE and MS, animal models of EAE have furthered our knowledge on the role of NOX in disease progression.

##### Neuroprotective effect of NOX inhibition in multiple sclerosis

The use of EAE animal models in conjunction with NOX inhibition techniques show a more causative role of NOX that may be contributing to MS pathology. In EAE in vitro studies, NOX inhibition with apocynin was able to reduce BBB permeability seen in EAE [301, 306]. Early studies have shown that blocking NOX via apocynin or DPI reduces ROS formation and prevents phagocytosis of myelin [307]. Similar results were obtained by either blocking NOX assembly or by deletion of p47phox, where ROS production and neuroinflammation were both attenuated in a model of isolated myelin and primary microglial culture [308]. Studies of the p47phox subunit report a complex function. Mice with p47phox deletion develop decreased EAE, showing no obvious signs of EAE as compared to WT mice who developed modest of severe disease after EAE induction [309]. However, mutant mice with truncated or nonfunctional p47phox proteins showed enhanced disease progression after EAE induction [310], indicating that the oxidative burst of NOX enzymes may serve a beneficial role in modulating disease progression. Nonetheless, more studies have reported benefits of NOX inhibition. Isolated microglia from NOX2 KO mice showed reduced toxicity to oligodendrocytes [295]. Inhibition with DPI also prevented activated microglia from killing oligodendrocytes [295]. In vivo, NOX2 deletion in mouse models of EAE prevented the weight loss, attenuated oligodendrocyte loss, and reduced microglia reactivity that were all observed in WT EAE mice [311]. Deletion of NOX2 also improved neurological outcome in EAE mice [311], showing that these NOX2 KO mice are more resistant to EAE. Mice treated with apocynin to inhibit NOX also showed reduced clinical symptoms of EAE, reduced demyelination, and reduced infiltration of immune cells [306]. It has also been shown that apocynin can reduce synaptic plasticity in the hippocampal CA1 region during a remission phase of EAE [305]. The evidence based on genetic studies of NOX deletion and inhibition demonstrates that modulation of NOX can therapeutically inhibit some clinical features and neuropathological changes associated with EAE, and ultimately MS.

### Translational relevance - NOX inhibitors as potential therapeutic agents

Although there is much promise in using a NOX inhibitor for the management of brain injury and neurodegenerative disorders, preclinical studies have been limited due to many factors. To date, preclinical ROS scavenging trials lack consistency in demonstrating their beneficial effects [312] despite showing promise in animal studies. The lack of translational success could possibly be due to issues with targeting within the therapeutic window, animal model adequacy, the sample size, and bioavailability [313]. The evidence discussed above supporting a role of NOX in brain injury and neurodegeneration has provided impetus towards examining the therapeutic efficacy of NOX inhibitors in these disorders [73], as this approach may offer the best therapeutic option for treating disorders associated with oxidative stress [314]. One challenge that NOX inhibitors must overcome for successful translation to the clinic is the timing of application. Most inhibition studies referenced in the above sections have been initiated before injury or during the acute phase of the disease immediately following injury. Not enough studies have been done in exploring whether delayed inhibition of NOX at later time points following injury may offer different therapeutic effects, especially in disease models where NOX activation post-injury is prolonged. Since NOX inhibition at later time points are more relevant to a clinical setting, more exploration in the early versus late inhibition can increase the success of translating NOX inhibitors to clinical models. Challenges also exist in determining the optimal treatment window for chronic neurodegenerative disorders that progress over many years, where no single injury, such as in stroke or TBI, is the root of disease. In chronic neurodegenerative diseases, NOX inhibition may aim to primarily modulate the chronic inflammation that exacerbates disease progress. Future studies utilizing NOX inhibitors should pay attention to the acute or chronic nature or studied disease models. In the sections below, we discuss the utility of various isoform-specific and non-specific NOX inhibitors, as well as more challenges that still need to be met for successful translation to the clinics.

#### Non-specific NOX inhibitors

##### Apocynin

Apocynin is perhaps the most studied drug of all the available NOX inhibitors. It was isolated from the medicinal plant *Picrorhiza kurroa* and inhibits NOX2 via preventing the membrane translocation of p47phox and p67phox [315–320], without interfering with physiological intracellular killing and defense mechanisms [319]. Apocynin also reduces oxidative damage by scavenging hydrogen peroxide and hypochlorous acid in phagocytic cells [150, 321]. Apocynin inhibition of NOX in neutrophils was reported to require myeloperoxidase [150]. Some potential advantages of apocynin as a potential therapeutic agent in neurodegenerative disorders include its reported low toxicity and ability to pass the BBB [203, 277, 322], and its documented ability in Phase I clinical studies to reduce oxidative stress after aerosol administration [317, 323]. A potential disadvantage of apocynin as a therapeutic agent could be its reported “off target” direct ROS scavenging effects. However, as mentioned previously, apocynin-induced neuroprotection against ischemic injury has been shown to be lost in NOX2 knockout animals [157], suggesting its neuroprotective effects are NOX2 dependent. In addition, as a therapeutic agent, having other ROS inhibiting effects such as scavenging ROS may not necessary be a negative, as multimodal ROS inhibition could actually lead to a greater efficacy and improved therapeutic outcome.

##### DPI

Diphenyleneiodonium chloride (DPI) has been used as an inhibitor of NOX in both in vivo and in vitro studies investigating oxidative stress in neurodegenerative disorders [145, 246, 247, 307]. DPI accepts an electron from flavin, thereby preventing the generation of ROS [324]. However, this mechanism of action allows DPI to inhibit other flavoenzymes. Thus, it is unlikely that DPI is selective for a specific NOX isoform. In addition to inhibiting NOX [325], DPI inhibits activities of NOS [326], xanthine oxidase, and cyp450 enzymes [246, 324], and blocks potassium and calcium currents [327], suggesting a nonselective ion channel blocking function that can protect neurons against glutamate excitotoxicity [328]. Despite its nonspecific action and high toxicity [329], at micromolar doses, DPI has been reported to inhibit NOX without any off target activities [330], opening the exploration of optimum dosage in evaluation of a drug’s specificity for NOX. Nonetheless, development of more targeted approaches using specific NOX inhibitors is considered most needed by the field, so as to avoid inhibition of all NOX isoforms, which could lead to increased risk of negative side effects in patients.

#### NOX2 specific inhibitors

##### Gp91ds-tat

Gp91ds-tat is reported to be a specific and efficacious peptide inhibitor that binds directly to NOX2 to impair its activity [249, 331]. Introduced in 2001, gp91ds-tat is a chimeric peptide that inhibits the association of p47phox with gp91phox and thus inhibits ROS production by NOX [249]. GP91ds-tat is specific for NOX2-containing gp91phox and not xanthine oxidase; however due to the homologous structure between NOX1 and NOX2, gp91ds-tat may also inhibit the assembly of NOX1 [249], as well as its activity [331]. In a cell free system, gp91ds-tat inhibits NOX activity up to 80%, and does not act as a scavenger of superoxide, but this inhibition is reduced to roughly 35% in intact human neutrophils [249], possibly due to the cell’s ability to overcome such an inhibition. Despite efficacy in animal models, gp91ds-tat has very limited oral bioavailability, and thus requires intravenous administration. It is also not clear whether it can pass the BBB.

##### GSK2795039

GSK2795039 is a novel, small molecule NOX2 inhibitor that has been shown to inhibit NOX2-dependent ROS generation. In vitro characterization defined GSK2795039 as a reversible, direct inhibitor of NOX2, while in vivo studies showed brain penetrance with systemic administration [332]. This compound has been tested in a mouse model of acute pancreatitis to show reduced pancreatic cell necrosis [332] paralleling results found in gp91^phox^ knock out mice [333]. However, preclinical studies are needed to test the efficacy of this novel small molecule NOX2 inhibitor in brain injury and neurodegenerative disorders.

#### NOX selective inhibitors

##### VAS2870

VAS2870 is a low molecular weight NOX inhibitor that can maintain basal ROS production in a model of endothelial cells [334] while decreasing ROS due to NOX. Though specific for NOX, VAS2870 does not appear to differentiate between specific NOX isoforms, including DUOX. Because of the variability in subunits amongst the NOX and DUOX enzymes, VAS2870 is thought to act on a common core flavocytochrome [335], but does not appear to inhibit other classes of flavoenzymes [70]. In an ischemic stroke animal model, administering VAS2870 intrathecally several hours after cerebral ischemia offered the same protection as deletion of NOX4, which was implicated as the source of ROS in this particular model [140]. However, VAS2870 has been reported to inhibit other NOX isoforms as well. For instance, in a liver tumor model, VAS2870 inhibited NOX1 [336], while it also inhibited DUOX-induced ROS in a model of zebrafish wound healing [337]. Additionally, there are some contradictions found in the mechanism of action of VAS2870. It has been cited to inhibit NOX2 in whole cell lysates [338], and even as a direct NOX2 inhibitor [339]; yet, another study suggests an inhibitory action upstream of NOX2 because the originally proposed NOX2 inhibition was not seen in a purified enzyme preparation [340]. A structurally similar analogue, VAS3947 shows higher solubility but is similarly nonspecific to the various NOX isoforms [70]. Though VAS2870 has been validated as an inhibitor of NOX, its lack of specificity to NOX isoforms, low solubility, and need for intrathecal route of administration make it unlikely to be successful for treatment of brain injury or neurodegenerative disorders in humans.

##### GKT-136901

Identified in 2010, GKT-136901 is a novel inhibitor of NOX that has high specificity for NOX1 and NOX4, with low affinity and partial inhibition of NOX2 [341], and low affinity and low to no inhibition of other ROS producing enzymes [341, 342]. This novel dual-inhibitor has also shown ability to scavenge peroxynitrite, which may prove useful therapeutically [343], but may confound studies utilizing GKT-136901 to investigate specific roles of NOX isoforms in disease pathology. GKT-136901 has high oral availability and in vivo plasma concentrations after administration [342], but its ability to pass the BBB is unclear. Since it is a selective NOX inhibitor and not single isoform specific, the potential for side effects may be increased.

##### GLX351322

A newly developed NOX inhibitor, GLX351322, is proposed to have relatively higher selectivity to NOX4 over NOX2. In a model of type 2 diabetes, GLX351322 treatment prevented ROS production and death in human islet cells [344]. Due to it only being recently developed, studies are needed to further confirm the specificity of this novel inhibitor and establish its ability to target NOX therapeutically in brain injury and neurodegenerative disorders.

### Translational challenges and emerging concepts

While we have learned much about the biology, function and targeting of NOX subunits in the last decade, much more needs to be known. Several major challenges remain that must be met in order to translate basic discoveries in the field to effective therapies in the clinic. In the next section, we summarize these challenges and some potential solutions and future directions that may help move the field forward.

#### The need for inducible, cell-type and NOX isoform specific knockout models

All of the mutant animal studies discussed in this review used global knockout animal models. While these studies have yielded important insights, global knockout models are not without limitations. For instance, it is often difficult to distinguish primary versus secondary effects in global KO models. Furthermore, the knockout is lifelong, including throughout development, which could cause developmental confound and/or compensation issues. Finally, in global knockout animals the NOX gene is knocked out in multiple different cell types, and thus one cannot easily determine the role of a particular NOX- expressing cell type in a specific process or disorder. To address these limitations, the field would benefit from development of inducible, cell-specific NOX subunit knockout approaches in animal models of brain injury and neurodegenerative disorders. With the recent advent of CRISPR-Cas9 and related gene editing technologies, knockout model generation has become more cost effective and less time consuming, which should spur more rapid progress toward this goal. In addition to use of this technology in mice, it would be advantageous to also extend the inducible, conditional NOX knockout animal development to rats, so as to further confirm results in a different species.

#### The challenge of developing specific NOX inhibitors that lack significant side effects

An additional major challenge to the field is how to develop NOX inhibitors that avoid “off target” and non-specific effects, and have reduced potential for negative side effects in patients. It has been suggested this may be achieved through development of isoform-specific inhibitors that target only a specific NOX isoform. Indeed, effort has already been devoted to development of such isoform-specific NOX inhibitors, which is expected to continue and grow. In addition to inhibitors, another possibility for isoform-specific NOX inhibition is siRNA knockdown. However, delivery of siRNAs to the brain is a challenge and requires more work and study before this would be a viable translational approach. Alternatively, a new approach that may yield effective isoform-specific NOX inhibitors is aptamer technology [345–348]. Aptamers are single-stranded nucleic acids that bind to their targets with high affinity and specificity and can directly block or interfere with the functions of the target proteins [348]. In addition, aptamers can be designed to penetrate the blood brain barrier [347, 349], can deliver additional therapeutic cargos (such as siRNA) to cells in a cell-type specific manner [346, 348, 350], and can be reversible [351]. Cell-type specific targeting could help avoid side effects by restricting inhibition to the major cell-type implicated in the pathology.

An additional therapeutic concern for most of the available NOX inhibitors is that they would inhibit *both* pathological and physiological NOX signaling. This is a significant concern, as inhibition of physiological NOX signaling could lead to significant side effects, especially when chronic NOX inhibition is utilized (which likely would be needed in chronic neurodegenerative disorders). Therefore, consideration must be given to strategies that would lead to interventions that would only inhibit pathological NOX signaling. This might be achieved by targeting critical interactions of NOX isoforms proteins with other proteins necessary for NOX to become active in pathological situations. Since aptamers have high specificity and affinity, and can be chemically modified to interfere with specific protein-protein interactions [352], aptamers technology might be one way to explore development of inhibitors that target only pathological NOX signaling. Furthermore, it is known that some NOX subunits require post-translational modification for activation [353]. Since aptamers can be designed to recognize post-translational modified sequences [348], they potentially could also be useful to inhibit post-translationally “activated” NOX subunits.

#### The challenge of balancing NOX activity for regenerative medicine

While NOX activation has a role in the pathology of brain injury and neurodegenerative disorders, it is currently unknown whether it plays any role in regeneration of the brain after injury. Intriguingly, it is known that there is an increase in adult neurogenesis following various brain insults, including stroke [232, 354], TBI [233, 234], and even in certain stages of neurodegenerative disorders such as AD [235, 236]. Furthermore, recent work has shown that NOX2 is colocalized in neural stem cells in the sub-ventricular zone, suggesting it may have a role in regulation of neurogenesis [241]. In support of this possibility, both in vivo and in vitro studies have recently shown that ROS generation and the activation of NOX enzyme are essential for the proliferation and self-renewal capacity of neural stem/progenitor cells [241, 242, 244, 245]. Under physiological conditions, it has been suggested that the ROS generation in the stem cell “niche” is mediated by NOX2, as (i) NOX2 expression was detected in neural stem cells and increased when these cells were stimulated with mitogen factors to promote proliferation [242, 245]; (ii) NOX2-deficient mice show decreased neurogenesis in the sub-ventricular zone and the dentate gyrus, two major regions well documented for adult neurogenesis [242, 244]; and (iii) cultured neural stem cells obtained from adult NOX2-deficient mice show decreased proliferation and self-renewal capacity [242, 244]. These studies only examined *basal* neurogenesis in a non-injury and non-neurodegenerative context. Given that brain injury can markedly elevate levels of ROS and NOX activation, additional studies are needed to examine whether NOX2-derived oxidative stress, influences the proliferation, self-renewal capacity, and cell-fate determination of both endogenous and exogenous neural stem cells following brain injury and in neurodegenerative disorders. Such studies could yield important new insights on the role of NOX in repair and regeneration of the brain, and help better inform future development strategies for therapeutic NOX intervention in brain injury and neurodegenerative disorders. Furthermore, the role of NOX in the vasculature needs to be considered when utilizing therapeutic NOX intervention. NOX enzymes are reported to promote angiogenesis: (i) NOX1 inhibition in endothelial cells reduce angiogenesis [355]; (ii) NOX4 is reported to have a protective role via enhancing pro-angiogenic signaling after hypoxia in cardiac tissue [356]; and (iii) BDNF induces NOX activation via p47phox phosphorylation promoted angiogenesis in vascular endothelial cells [357]. There may also be differential regulation of NOX isoforms in the vasculature in response to different neurodegenerative disorders, which may lead to variability in the potentially negative effects of utilizing non-selective NOX inhibitors. NOX inhibition has also been reported to affect synaptic plasticity and LTP induction [358]. Potential negative effects on the cerebrovasculature and neuroplasticity need to be considered when using NOX inhibitors. Despite potential pitfall of NOX inhibitors to alter physiological processes, it is important to note that apocynin has been used in clinical trials with low observed toxicity and no significant side effects [317, 323], supporting the continued pursuit of NOX inhibitors as potential therapy for neurodegenerative disorders.

## Conclusions

There is significant evidence that NOX subunits and NOX activity are elevated in various neurodegenerative disorders, and that genetic ablation and pharmacological inhibition studies support the possibility of targeting NOX subunits for therapeutic benefit in neurodegenerative disorders. However, clearly there still remain many challenges ahead for the discovery of effective and NOX-specific targeting that would yield benefit in neurodegenerative disorders while minimizing negative side effects and generating a healthy neuronal environment for neurogenesis. While addressing these challenges remains problematic, the continued advancement of technology offers hope that realization of this goal may be achievable in the near future.

## Abbreviations

AD: Alzheimer’s disease
ALS: Amyotrophic lateral sclerosis
Aβ: Beta-amyloid
CNS: Central nervous system
DNA: Deoxyribonucleic acid
DPI: Diphenyleneiodonium chloride
DUOX: Dual oxidase
EAE: Experimental autoimmune encephalomyelitis
FAD: Flavin adenine dinucleotide
HD: Huntington’s disease
JNK: Jun-N-terminal kinase
MAPK: Mitogen-activated protein kinases
MS: Multiple sclerosis
NADPH: Nicotinamide adenine dinucleotide phosphate
NFκb: Nuclear factor-kappa-b
NOX: NADPH oxidase
NOXA1: NADPH oxidase activator 1
NOXO1: NADPH oxidase organizer 1
PD: Parkinson’s disease
phox: Phagocytic oxidase
PI3K: Phosphoinositide-3 kinase
PKA: Protein kinase A
PKB: Protein kinase B
PKC: Protein kinase C
pMCAO: Permanent middle cerebral artery occlusion
RNS: Reactive nitrogen species
ROS: Reactive oxygen species
TBI: Traumatic brain injury
tMCAO: Transient middle cerebral artery occlusion
